# A novel method of fuzzy fault tree analysis combined with VB program to identify and assess the risk of coal dust explosions

**DOI:** 10.1371/journal.pone.0182453

**Published:** 2017-08-09

**Authors:** Hetang Wang, Jia Li, Deming Wang, Zonghou Huang

**Affiliations:** 1 Key Laboratory of Coal Methane and Fire Control (China University of Mining and Technology), Ministry of Education, Xuzhou, China; 2 School of Safety Engineering, China University of Mining and Technology, Xuzhou, China; 3 School of Environment Science and Spatial Informatics, China University of Mining and Technology, Xuzhou, China; Southwest University, CHINA

## Abstract

Coal dust explosions (CDE) are one of the main threats to the occupational safety of coal miners. Aiming to identify and assess the risk of CDE, this paper proposes a novel method of fuzzy fault tree analysis combined with the Visual Basic (VB) program. In this methodology, various potential causes of the CDE are identified and a CDE fault tree is constructed. To overcome drawbacks from the lack of exact probability data for the basic events, fuzzy set theory is employed and the probability data of each basic event is treated as intuitionistic trapezoidal fuzzy numbers. In addition, a new approach for calculating the weighting of each expert is also introduced in this paper to reduce the error during the expert elicitation process. Specifically, an in-depth quantitative analysis of the fuzzy fault tree, such as the importance measure of the basic events and the cut sets, and the CDE occurrence probability is given to assess the explosion risk and acquire more details of the CDE. The VB program is applied to simplify the analysis process. A case study and analysis is provided to illustrate the effectiveness of this proposed method, and some suggestions are given to take preventive measures in advance and avoid CDE accidents.

## Introduction

Coal dust, produced in coal mining activities, can lead to coal dust explosions (CDE), posing a serious threat to miners' occupational safety [1, 2]. CDE are a major disaster accident in coal mines, often causing heavy casualties and huge economic losses [3, 4]. In China, over 85% of underground coal mines face the risk of coal dust explosion and the number of casualties from CDE exceeded 5000 between 1949 and 2015 [5–7]. In September 2000, an extremely large CDE, caused by a gas explosion, occurred in Muchonggou coal mine in Guizhou province and led to 162 deaths and a direct economic loss of 12 million yuan [8]. In May 2004, another shocking CDE occurred in Dongfeng coal mine in Heilongjiang province, leading to the deaths of 171 miners [5]. In recorded history, the most serious CDE occurred in 1942 in Benxi coal mine, which caused the deaths of 1549 miners and left 246 injured [7]. The damage caused by the CDE is destructive and the current technique is incapable of avoiding the destruction when a CDE occurs, making an early warning system extremely important. With this concern, an effective method for identifying and assessing the risks of CDE in advance is very necessary. By identifying the causes of CDE and assessing the probability of the various causes and the CDE, we can determine the weak links of the coal mine system and take preventive measures in advance to avoid occurrences of CDE.

However, few studies have been done to investigate a scientific assessment of CDE risk and obtaining the exact probability data of each basic event is almost impossible, which limits the application of conventional fault tree analysis (FTA), due to the complexity and fuzziness of coal mine environment. Fuzzy mathematics is an effective method employed to solve problems with fuzzy characteristics [9]. For example, fuzzy theory was employed to analyze pricing and retail service decisions in fuzzy uncertainty environments [10] and an optimistic decision-making method was proposed for optimization problem based on fuzzy mathematics [11]. This paper employs FTA, which is a powerful technique used for evaluating system reliability or accidents in other industrial fields [12–15], to assess CDE risk. In addition, VB program is also employed to overcome the difficulty of large amount of computation and artificial mistakes in the quantitative analysis process, which limits the direct application of conventional FTA.

Under this background, the authors proposed a novel method combined fuzzy set theory and VB program for identifying and assessing CDE risk, which could play an important role in the prevention of CDE accidents. In this paper, we first gave a brief introduction to the method of fault tree analysis combined with fuzzy set theory and constructed the fault tree for CDE. Then the qualitative and quantitative analysis of the CDE fault tree was conducted. Subsequently, the VB program was proposed as a tool to simplify the analysis procedure and promote analysis efficiency. Finally, a case study was carried out to verify the effectiveness of the proposed novel method.

## General idea and construction of CDE fault tree

### General procedure of the novel method

In this paper, fuzzy set theory is introduced to overcome the restrictions of conventional FTA. Fault tree analysis combined with fuzzy set theory has been proven to be effective on solving such problems [16, 17], and some researchers have applied this method to analyze some accidents [18, 19].

**Fig 1:**
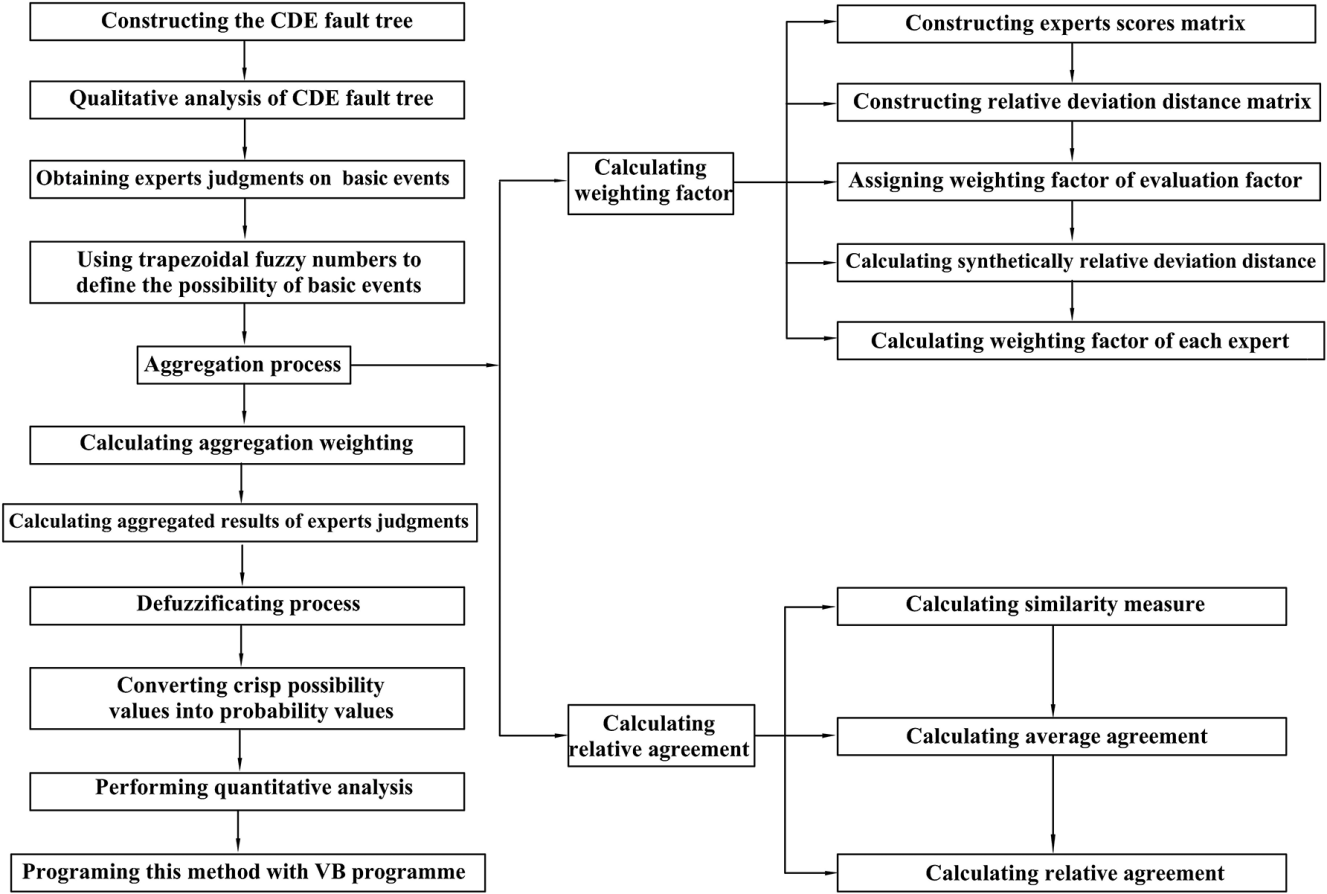
The procedure and principle of the proposed method.

The procedure and the principle of the proposed methodology are presented in Fig 1. Based on the explosion mechanism and influence factors, a fault tree for CDE can be constructed, and then some arithmetic operations of intuitionistic trapezoidal fuzzy numbers will be used to calculate the failure probability data for basic events, which are expressed as intuitionistic trapezoidal fuzzy numbers by the expert elicitation. With the exact probability data of each basic event, the further quantitative analysis for the FTA of CDE can be carried out. In addition, the Visual Basic (VB) program is proposed to overcome the inherent drawback of conventional FTA, which not only analyzes the risk quickly but also reduces artificial mistakes. At last, a case study is implemented to illustrate the effectiveness of this proposed method.

### Construction of CDE fault tree

**Fig 2:**
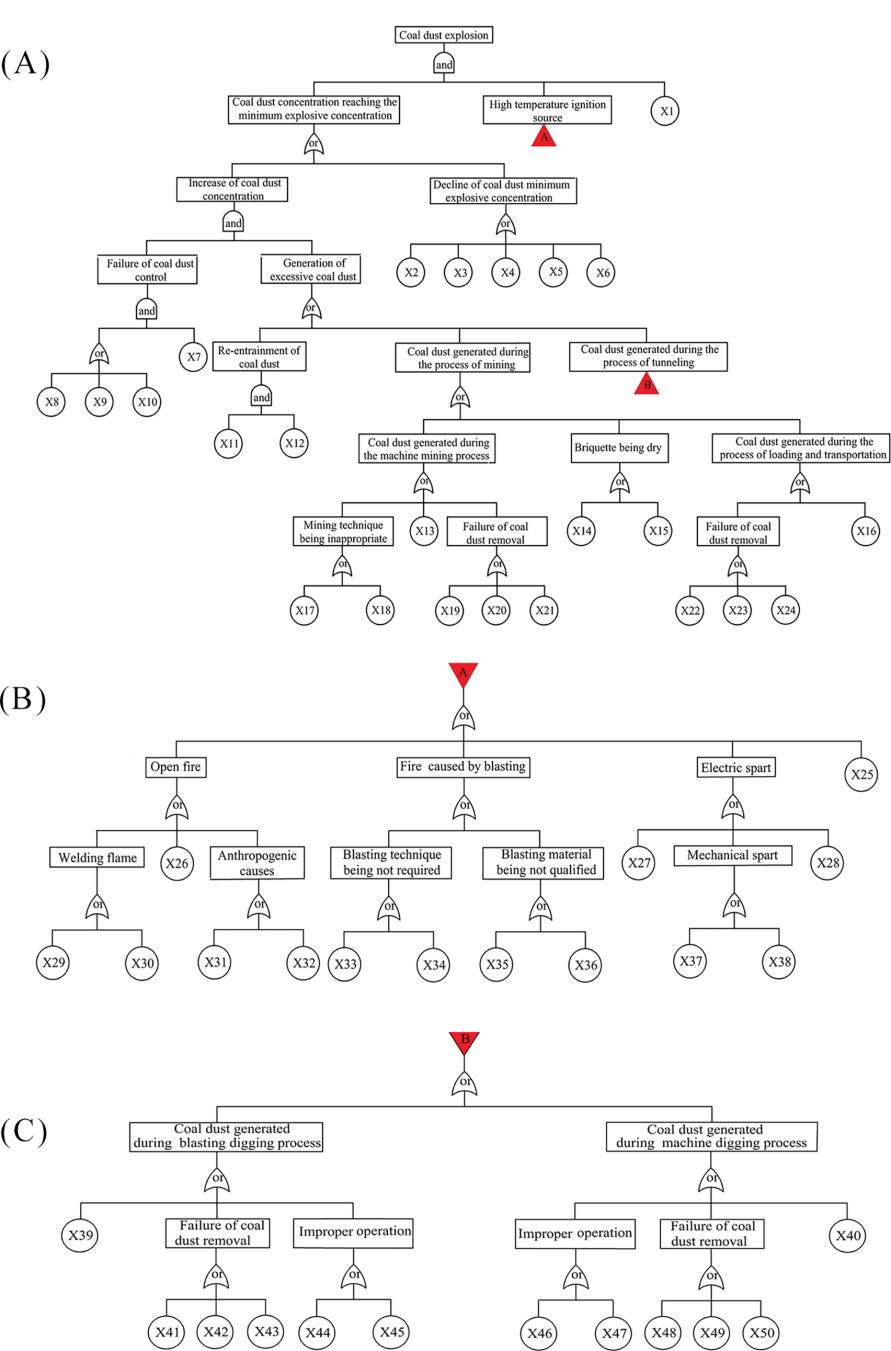
Schematics of the CDE fault tree. (A) General diagram; (B) Details of intermediate event A; (C) Details of intermediate event B.

FTA is a deductive and powerful method for evaluating coal mine system safety and identifying its potential causes that lead to undesired CDE. In this paper, the fault tree starts with the CDE and work backwards towards three intermediate events that must occur together: 1. “the concentration of coal dust reaching the minimum explosive concentration”, 2. “high temperature ignition source”, and 3. “the coal dust being explosive”. We continue to develop the fault tree until all branches have been terminated by 50 basic events, which are shown in the Table 1. Finally, a complete CDE fault tree is constructed, as shown in Fig 2A, 2B and 2C.

**Table 1:** Basic events of the CDE fault tree.

Notations	Meanings
X1	Coal dust being explosive
X2	Increase of concentration of coal dust
X3	Shortening of coal dust particle size
X4	Reduction of ash in the coal dust
X5	Increase of coal dust volatility
X6	Reduction of moisture in the air
X7	Testing personnel being not responsible
X8	Inappropriate operations of equipment for coal dust removal of related operators
X9	Malfunction of equipment for coal dust removal
X10	Low quality of equipment for coal dust removal
X11	Force of lifting coal dust underground generated by explosion
X12	Accumulation of much coal dust underground
X13	Taking no measures to remove coal dusk in the process of machine mining
X14	No water injection into coal mass before machine mining
X15	Poor effect of water injection into the coal mass
X16	Taking no measures to remove coal dusk in the process of loading and transportation
X17	Inappropriate equipment used for machine mining
X18	Inappropriate operations of mining machines operators
X19	The inappropriate operations of equipment for coal dust removal of mining machines operators
X20	Malfunction of equipment for coal dust removal in the process of machine mining
X21	Low quality of equipment for coal dust removal in the process of machine mining
X22	Inappropriate operations of equipment for coal dust removal of loading and transportation operators
X23	Malfunction of equipment for coal dust removal in the process of loading and transportation
X24	Low quality of equipment for coal dust removal in the process of loading and transportation
X25	Fire caused by friction
X26	Fire generated by gas explosion
X27	Inappropriate operations of equipment
X28	Short-circuit line
X29	Inappropriate operations of welders
X30	Low quality of equipment for welding
X31	Workers not obeying the rulers
X32	Inappropriate use of miners’ lamp
X33	Fault operation of blasting operators
X34	Inappropriate operations of blasting operators
X35	Low quality of blasting material
X36	Loss of efficacy of blasting material
X37	Malfunction of equipment
X38	Low quality of equipment
X39	Taking no measures to remove coal dusk in the process of blasting mining
X40	Taking no measures to remove coal dusk in the process of machine digging
X41	Inappropriate operations of equipment for coal dust removal of blasting mining operators
X42	Malfunction of equipment for coal dust removal in the process of blasting mining
X43	Low quality of equipment for coal dust removal in the process of blasting mining
X44	Inappropriate equipment used for blasting mining
X45	Inappropriate operations of blasting mining operators
X46	Inappropriate operations of equipment for coal dust removal of machine digging operators
X47	Malfunction of equipment for coal dust removal in the process of machine digging
X48	Low quality of equipment for coal dust removal in the process of machine digging
X49	Inappropriate equipment used for machine digging
X50	Inappropriate operations of machine digging operators

## Qualitative and quantitative analysis of the CDE fault tree

### Qualitative analysis

Aiming to identify the minimal cut sets (MCSs) and the minimal path sets (MPSs), which are the undeveloped combinations of basic events, qualitative analysis is performed. CDE will occur if basic events in one MCS fail together, and we can identify various paths that lead to the CDE occurrence by MCSs. Once the CDE occur, it is convenient to identify the causes with better understanding of MSCs. Besides, the most critical MCS, which leads to the occurrence of CDE with greatly possibility, can be identified and some effective measures will be proposed based on the MCSs. In contrast, the CDE will not occur if basic events in one MPS never fail together and the MPSs can help us discover preventive measures. With the help of quantitative analysis, preventing the occurrence of basic events in the MPS that its occurrence possibility is the highest can be adopted as the main preventive method, which can prevent the occurrence of CDE with high performance. By using a combination of the Fussell-Vesely algorithm and the rules of Boolean algebra [20, 21], MCSs and MPSs can be obtained from Eq (1) and Eq (2), respectively:

$$\begin{array}{l} T=MCS1+MCS2+\cdots \cdots +MCS1119+MCS1120\\ \;=\sum_{n=25}^{38}\sum_{j=8}^{10}X1X7X11X12XnXj+\sum_{n=25}^{38}\sum_{j=8}^{10}\sum_{k=13}^{24}X1X7XnXjXk+\\ \;=\sum_{n=25}^{38}\sum_{j=8}^{10}\sum_{g=39}^{50}X1X7XnXjXg+\sum_{n=25}^{38}\sum_{m=2}^{6}X1XnXm \end{array}$$ (1)

$$\begin{array}{l} T^{\prime }=MPS1+MPS2+MPS3+MPS4+MPS5+MPS6\\ \;=\prod_{n=2}^{7}Xn^{\prime }+\prod_{n=2}^{6}\prod_{j=8}^{10}Xn^{\prime }Xj^{\prime }+\prod_{n=2}^{6}\prod_{j=13}^{24}\prod_{k=39}^{50}X11^{\prime }Xn^{\prime }Xj^{\prime }Xk^{\prime }\\ \;+\prod_{n=2}^{6}\prod_{j=12}^{24}\prod_{k=39}^{50}Xn\prime Xj\prime Xk\prime +\prod_{n=27}^{38}X13^{\prime }X25^{\prime }Xn^{\prime }+X13\prime \end{array}$$ (2)

### Quantitative analysis

#### Trapezoidal fuzzy numbers to define the possibility of basic events

Conventional FTA is completely understood by the basic events represented by exact values of failure probabilities. However, exact values of the failure probabilities are difficult to obtain due to the physical constraints. To overcome this limitation, fuzzy set theory is employed. The concept of fuzzy set was introduced by Zadeh [22], and fuzzy set theory is widely used to deal with imprecise and vague information. In this paper, the probability of basic events for the CDE fault tree is described by the intuitionistic fuzzy numbers from a quadruple (a_1_,a_2_,a_3_,a_4_).

**Fig 3:**
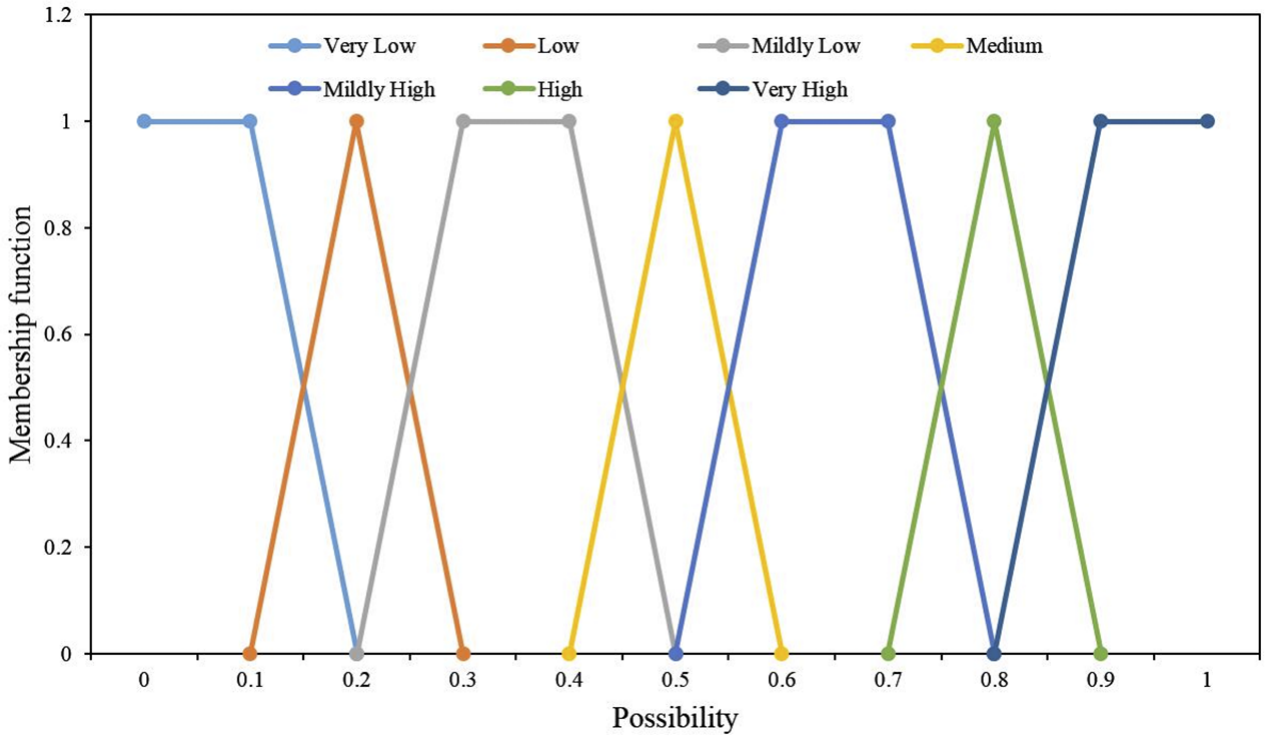
Fuzzy numbers representing linguistic variables.

Aiming to obtain the corresponding intuitionistic fuzzy numbers, we need to incorporate expert judgment into the FTA study. The expert elicitation method involves the direct estimation of probability by specialists in relevant fields, and the estimated failure probability values are closer to the real values. When judging the probability of basic events, experts usually give the judgment language: “equally”, “high”, “low”, and so on. Therefore, we need to convert linguistic terms into corresponding fuzzy numbers. The corresponding membership function is shown in Fig 3.

#### Aggregation stage

In this paper, three experts in relevant fields are invited to judge the possibility of basic events occurring. As they are with different backgrounds, the opinions of the experts are different for the same basic events and it is necessary to aggregate the opinion of each expert to reach a consensus. The aggregation stage can be divided into four steps.

#### Step 1 Weighting factor calculation

Each expert expresses his opinions of the basic events based on his/her background: professional experience, educational or technical qualification, and professional position. The rating of these judgments is necessary due to the difference in background factors. The weighting scores of experts are defined as shown in Table 2. In this paper, synthetically relative deviation distance was used to calculate the weighting factor for each expert.

**Table 2:** Weighting scores of different experts.

Constitution	Classification	score
Professional position	Professor, GM/DGM, Chief Engineer, Director	5
	Assistant, Professor, Manager, Factory Inspector	4
	Engineer, Supervisors	3
	Foreman, Technician, Graduate apprentice	2
	Operator	1
Professional experience(years)	> = 20	5
	15 to 19	4
	10 to 14	3
	5 to 9	2
	<5	1
Educational or technical qualification	Ph. D or M. Tech	5
	MSc or B. Tech	4
	Diploma or BSc	3
	ITI	2
	Secondary school	1

*a*. *The construction of the expert scores matrix*

Definition 1: Let u_ij_ be the score of expert i (i = 1,2,3) on item j (j = 1,2,3), where “item 1” represents the “Professional position”, “item 2” represents the “Professional experience (years)”, and “item 3” represents the “Educational or technical qualification”.

Then we can acquire the expert scores matrix as follows:

$$\left[\begin{array}{ccc} u_{11} & u_{12} & u_{13}\\ u_{21} & u_{22} & u_{23}\\ u_{31} & u_{32} & u_{33} \end{array}\right]$$

*b*. *The construction of the relative deviation distance matrix*

Definition 2: Let δ_ij_ be the relative deviation distance of item j (j = 1,2,3) of expert i (i = 1,2,3)

Then, *δ*_*ij*_ can be obtained by Eq (3):

$$\delta_{\mathrm{ij}}=\frac{\left|u_{\mathrm{imax}}-u_{\mathrm{ij}}\right|}{u_{\mathrm{imax}}-u_{\mathrm{imin}}}\;\left(i=1,2,3\;j=1,2,3\right)$$ (3)

Where u_imax_ = max{u_1i_,u_2i_,u_3i_}, and u_imin_ = min{u_1i_,u_2i_,u_3i_}

Then the relative deviation distance matrix can be written as shown:

$$∆=\left[\begin{array}{ccc} \delta_{11} & \delta_{12} & \delta_{13}\\ \delta_{21} & \delta_{22} & \delta_{23}\\ \delta_{31} & \delta_{32} & \delta_{33} \end{array}\right]$$

Where Δ represents the relative deviation distance matrix.

*c*. *Assigning weighting factor of each evaluation factor*

It is necessary to consider the relative worthiness of each evaluation factor because one factor may show more importance over another one in reality. In this paper, the weighting factor of each evaluation factor is assigned by an expert who is responsible and extremely familiar with the system and is written in the following form:

$$A=\left(b_{1},b_{2},b_{3}\right)$$

Where *b*_*j*_ represents the weighting factor of item j (j = 1,2,3).

*d*. *The calculation of the synthetically relative deviation distance*

The synthetically relative deviation distance can be calculated from Eq (4):

$$d_{i}=d_{i}\left(u_{i},u^{0}\right)={\left(\sum_{j=1}^{m}{\left(b_{j}.\delta_{\mathrm{ij}}\right)}^{2}\right)}^{\frac{1}{2}}\;\left(j=1,2,3\right)$$ (4)

Where d_i_ represents the synthetically relative deviation distance of expert i (i = 1,2,3).

*e*. *The calculation of the weighting factor of each expert*

The weighting factor of each expert can be obtained using Eq (5):

$$D\left(i\right)=\frac{d_{1}+d_{2}+d_{3}-d_{i}}{2\left(d_{1}+d_{2}+d_{3}\right)}\;i=1,2,3$$ (5)

Where D(i) represent the weighting factor of expert i (i = 1,2,3).

#### Step 2 Relative agreement calculations

Due to the range of different backgrounds, the opinions of each expert have different weighting factors. To ensure that the opinions of all expert are taken into consideration, a relative agreement calculation is necessary during the aggregation weighting calculation process. The relative agreement calculation process is divided into three steps:

(1) Similarity measure calculation

This step calculates the similarity measure of two opinions on a same basic event. The opinions are described by a set of fuzzy numbers. In this paper, the similarity is obtained by calculating the arithmetic average minimum similarity degree, as expressed in Eq (6).

$$\varphi_{\left(i,j\right)}=\frac{2\sum_{i=1}^{3}\min\left[\mu_{i}\left(k\right),\mu_{j}\left(k\right)\right]}{\sum_{i=1}^{3}\left[\mu_{i}\left(k\right)+\mu_{j}\left(k\right)\right]}\;\left(k=1,2,3,4\right)$$ (6)

Where φ_(i,j)_ represents the similarity measure of expert i (i = 1,2,3) and expert j (j = 1,2,3) on the same basic event, μ_i_(k) represents the k-th number of the trapezoidal fuzzy number of expert i (i = 1,2,3), and μ_i_(k) represents the k-th number of the trapezoidal fuzzy number of expert j (j = 1,2,3).

(2) Average agreement calculation

The average agreement (AA) can be obtained by Eq (7):

$$\mathrm{AA}\left(i\right)=\frac{\sum_{\begin{array}{c} j=1\\ i\neq j \end{array}}^{3}\varphi_{\mathrm{ij}}}{2}$$ (7)

Where AA(i) represents the average agreement of expert i (i = 1,2,3).

(3) Relative agreement calculation

The relative agreement (RA) of each expert is calculated by Eq (8):

$$\mathrm{RA}\left(i\right)=\frac{\mathrm{AA}\left(i\right)}{\sum_{i=1}^{n}\mathrm{AA}\left(i\right)}\;\left(i=1,2,3\right)$$ (8)

Where RA(i) represents the relative agreement of expert i (i = 1,2,3).

#### Step 3 Aggregation weighting (AW) calculation

To balance the weighting factor and relative agreement, the aggregation weighting is calculated by Eq (9):

AW(i)=αD(i)+(1−α)RA(i)(i=1,2,3) (9)

Where AW(i) represents the aggregation weighting of expert i (i = 1,2,3), α represents the value of the relaxation factor of this proposed method and shows the importance of D(i) over RA(i).

#### Step 4 Calculation of the aggregated results of experts’ judgment (EG)

Following the above steps, we easily acquire a set of aggregated fuzzy numbers describing the probability of basic events and the aggregated results can be calculated using Eq (10):

$$\mu_{\mathrm{EG}}=\mathrm{AW}\left(1\right)\times \mu_{1}+\mathrm{AW}\left(2\right)\times \mu_{2}+\mathrm{AW}\left(3\right)\times \mu_{3}$$ (10)

Where *μ*_*EG*_ represents the aggregated results of the experts’ judgment, and *μ*_*i*_ represents the judgment of expert i (i = 1,2,3).

#### Defuzzification process

In this paper, in order to obtain quantifiable results about the probability of basic events, the center of area defuzzification technique [21, 23] is employed to defuzzify the fuzzy numbers. Defuzzification of a trapezoidal fuzzy numbers μ = (*a*_1_,*a*_2_,*a*_3_,*a*_4_) can be realized from Eq (11):

$$\begin{array}{l} P^{*}=\frac{\int_{a_{1}}^{a_{2}}\frac{x-a_{1}}{a_{2}-a_{1}}\mathrm{xdx}+\int_{a_{2}}^{a_{3}}\mathrm{xdx}+\int_{a_{3}}^{a_{4}}\frac{a_{4}-x}{a_{4}-a_{3}}\mathrm{xdx}}{\int_{a_{1}}^{a_{2}}\frac{x-a_{1}}{a_{2}-a_{1}}\mathrm{dx}+\int_{a_{2}}^{a_{3}}\mathrm{dx}+\int_{a_{3}}^{a_{4}}\frac{a_{4}-x}{a_{4}-a_{3}}\mathrm{dx}}\\ \;=\frac{1}{3}\frac{{\left(a_{4}+a_{3}\right)}^{2}-a_{4}a_{3}-{\left(a_{1}+a_{2}\right)}^{2}+a_{1}a_{2}}{\left(a_{4}+a_{3}-a_{1}-a_{2}\right)} \end{array}$$ (11)

Where P* represents the crisp possibility values of a basic event.

#### Converting crisp possibility values into probability values

The probability values (P) can be obtained from the possibility in Eq (12) [24]:

$$P=\{\begin{array}{cc} \frac{1}{10^{m}} & P^{*}\neq 0\\ 0 & P^{*}=0 \end{array}m={\left(\frac{1-P^{*}}{P^{*}}\right)}^{\frac{1}{3}}\times 2.301$$ (12)

#### Quantitative calculation

By the application of fuzzy set theory, the occurrence probability of basic events can be evaluated and the quantitative analysis can be carried out. In this paper, the occurrence probability of CDE, the occurrence probability of MCS and MPS, and the importance of each basic event are calculated.

1. The occurrence probability of CDE calculation (CDEOP)
  We can easily calculate the CDEOP when acquiring the occurrence probability of each basic event. We know the situation of CDE in this coal mine by the occurrence probability. We can also decide whether to take measures to avoid CDE immediately.
2. The occurrence probability of MCS and MPS calculation
  The occurrence probability of MCSs and MPSs is very important in the decision of targeted measures. By calculating the occurrence probability of MCSs (MCSOP), we can acquire the most crucial MCSs for the undesired CDE and take effective measures to avoid the CDE. By calculating the occurrence probability of MPSs (MPSOP), we can take the relative solution to avoid the CDE.
3. The importance of basic event calculation (IOBE)
  The IOBE calculation is employed to evaluate the contribution of each basic event to the occurrence of CDE. Using this method, we determine the relative important basic events and take corresponding measures to avoid the occurrence of these basic events, which will avoid the CDE effectively. The IOBE can be acquired by calculating the occurrence probability of the CDE while the occurrence probability of this basic event is considered 0, shown as Eq (13):
  $$i.\;PI_{j}=P_{P_{i}=0}$$ (13)
  Where *PI*_*j*_ represents the value of the importance of basic event i.
  Obviously, the smaller *PI* is, the more important basic event j (j = 1,2,3) is.

## Rapid assessment by the VB program

The CDE fault tree includes fifty basic events and we can acquire 1120 MCSs, so we need to employ a computer program to simplify the analysis process. VB program is a simple and convenient tool used for small software programs. In addition, it is convenient to download and install the VB program. This paper employs VB program to simplify the analysis process.

The program not only realizes the calculation of the CDEOP, MCSOP, MPSOP, and IOBE, but also realizes the ranking of each MCSOPs. Due to the actual requirements, the program ranks the top 10 MCSs consisting of 3 basic events, 5 basic events, and 6 basic events, respectively. In the program, MCSOP3 represents the occurrence probability of MCSs consisting of 3 basic events, MCSOP5 represents the occurrence probability of MCSs consisting of 5 basic events, and MCSOP6 represents the occurrence probability of MCSs consisting of 6 basic events.

**Fig 4:**
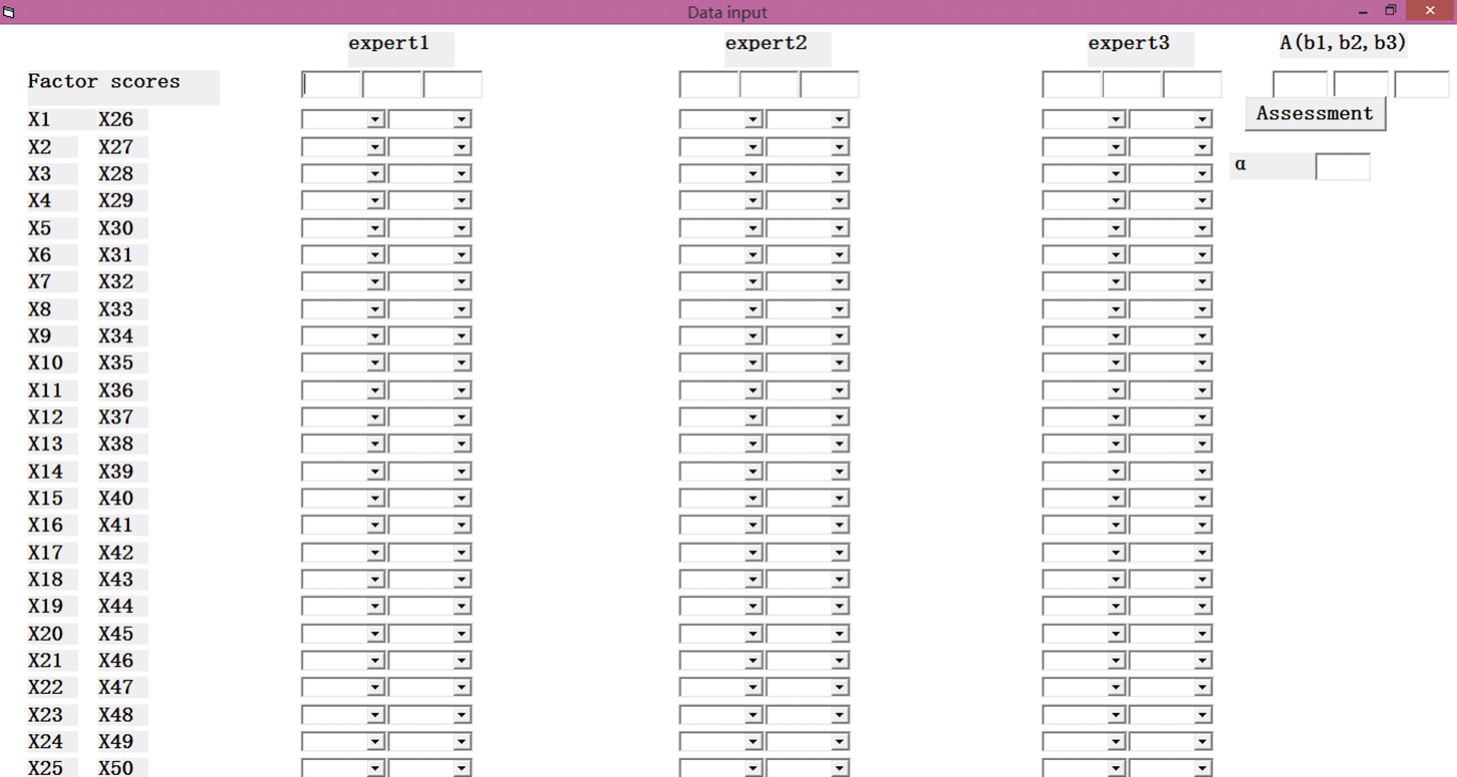
The initial input interface of the VB program.

The form of the VB program is shown in Fig 4.

## Case study

To illustrate the proposed method, the Zhuxianzhuang Coal Mine, which has a risk of CDE, is taken as an example.

### Background

The Zhuxianzhuang Coal Mine is located in Huaibei mining area, eastern China. It is a modern large scale mine with a 2.45 million tones production capacity. There are hundreds of miners working underground within a working shift, and there are three villages, a primary school, and a secondary school around this coal mine. If one CDE event occurs, there will be catastrophic results. Therefore, it is necessary to analyze the potential danger of a CDE for this coal mine.

### CDE risk identification and assessment

#### Step 1. Experts’ profiles

As mentioned above, three experts from different backgrounds are invited to evaluate the occurrence probability of basic events and the weights of the experts are not equal (Table 2). In order to calculate the weighting factor for each expert, a profile of each expert is necessary. Experts’ profiles and corresponding factor scores are shown in Table 3.

**Table 3:** Experts’ profiles and corresponding scores.

Expert serial number	Professional position/score	Professional experience(years)/score	Educational or technical qualification/score
1	Professor/5	13/3	Doctorate/5
2	Engineer/3	16/4	MSc/4
3	Operator/1	21/5	Secondary school/1

#### Step 2. Experts’ judgments

Experts’ judgments on the basic events are shown in Table 4.

**Table 4:** The judgments of the three experts.

NTS	EP1	EP2	EP3	NTS	EP1	EP2	EP3	NTS	EP1	EP2	EP3
X1	H	VH	H	X18	ML	ML	ML	X35	L	L	ML
X2	H	MH	H	X19	ML	L	VL	X36	L	L	L
X3	L	L	L	X20	M	ML	M	X37	L	ML	ML
X4	L	VL	VL	X21	VL	L	VL	X38	L	ML	ML
X5	L	ML	VL	X22	M	L	MH	X39	VH	VH	VH
X6	H	H	VH	X23	MH	H	M	X40	H	H	H
X7	H	H	VH	X24	L	L	ML	X41	H	H	VH
X8	H	VH	H	X25	H	H	VH	X42	M	M	L
X9	M	M	ML	X26	M	ML	ML	X43	L	L	ML
X10	L	L	VL	X27	H	H	VH	X44	L	L	ML
X11	H	H	VH	X28	L	M	ML	X45	H	H	VH
X12	VH	VH	H	X29	H	H	VH	X46	L	L	VL
X13	L	L	ML	X30	M	M	ML	X47	L	VL	M
X14	L	L	L	X31	VH	VH	VH	X48	L	L	ML
X15	VL	L	ML	X32	M	M	ML	X49	L	ML	L
X16	M	ML	ML	X33	M	MH	L	X50	M	M	ML
X17	ML	L	L	X34	M	M	M				

#### Step 3. Assigning the values of α, b_1_, b_2_, and b_3_

The exact values of α, b_1_, b_2_, and b_3_ are different for different coal mine systems, and it is necessary to assign values for α, b_1_, *b*_2_, *and b*_3_ before the analysis is performed.

Based on these experts’ suggestions, the value of α is 0.8, and *A* = (0.2,0.6,0.2).

#### Step 4. Data input

**Fig 5:**
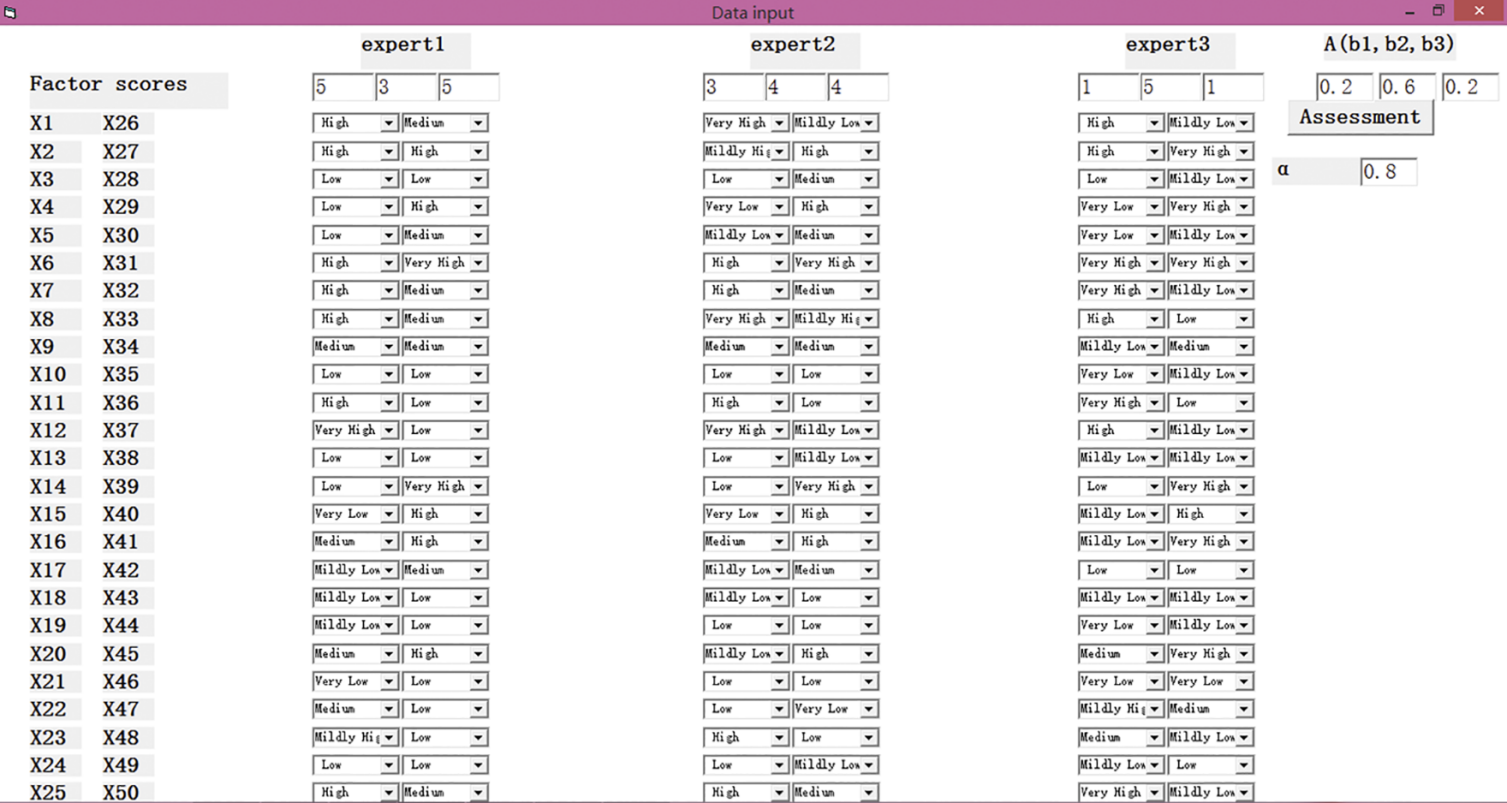
The interface of the VB program after data input.

Before the VB program analysis, we need to input the relevant data. Fig 5 shows the form of the VB program after data input.

#### Step 5. The results of the VB program analysis

**Fig 6:**
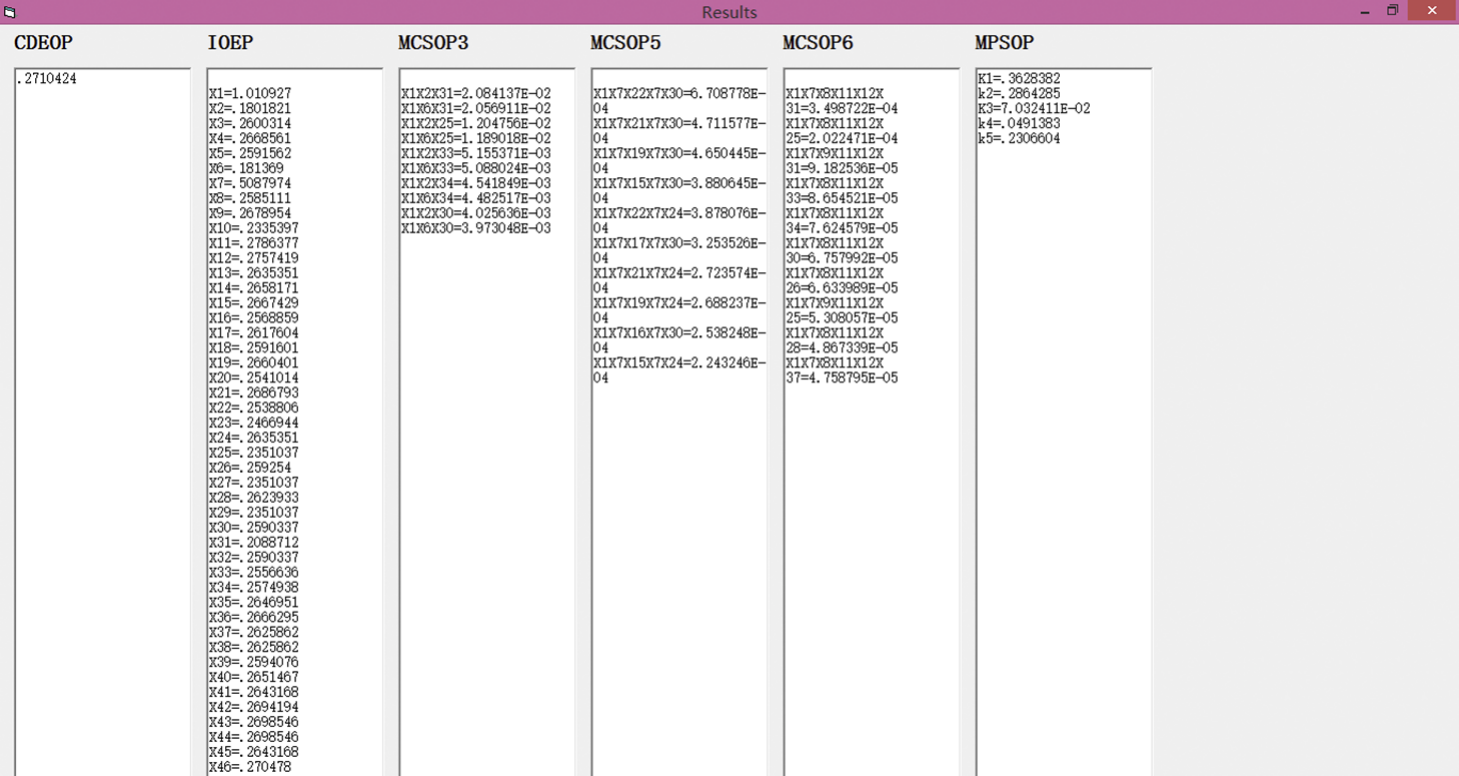
The results output interface of the VB program.

The results of the VB program analysis are shown in Fig 6.

Based on the IOEP values, we find that the most important basic event is X2 and the least important basic event is X7. The top 6 basic events are X2, X6, X10, X23, X25, and X31. From the occurrence possibility values of MCSs, we know that the MCSs of X1, X2, X31 and X1, X6, X31 are more critical. Based on the values of MPSOPs, MPS1 and MPS2 are the better choices for avoiding the occurrence of CDE.

### Suggestions

From the results of the VB program analysis, the following suggestions are provided:

1. It is better to take measures to avoid the occurrence of basic events X2, X6, X10, X23, X25 and X31. The IOEP values of above basic events were smaller relatively, which represents that these basic events are the dominant factor resulting in the CDE. Besides, the MCSs of (X1, X2, X31) and the MCSs of (X1, X6, X31) were the main ways leading to CDE. As long as one of the basic events in the MCS does not occur, this MCS will not contribute to the occurrence of CDE. We cannot change the explosiveness of coal dust, so the occurrence of X1 cannot be prevented. Hence, preventing the occurrence of these basic events can reduce occurrence possibility of CDE greatly. Blasting workers should obey the blasting rules and operate correctly. Technicians should take measures to control the moisture in the air. In addition, some necessary measures should be taken to avoid friction fire and prevent gas explosion. And managers should educate the workers about safety and the significance of correctly operating the equipment. They also need to clean up the settled coal dust in underground roadways in time.
2. When taking prevention measures, the MPS of MPS1 and MPS2 are the better choices in our opinion. Because the occurrence probability of the MPS1 and MPS2 are larger than others, the prevention measures from the MPS1 and MPS2 are more effective and easily realized. As long as all the basic events in the MPS do not occur, the CDE will not occur. Hence, some necessary measures should be taken to prevent the occurrence of X2, X3, X4, X5, X6, X7, X8, X9 and X10.

### Comparison with safety checklist analysis

The conventional method to evaluate the CDE risk is safety checklist analysis that experts judge the condition of potential risk listed the checklist. This evaluation method is simple and easy to master. However, this method is only used for simple qualitative analysis and cannot be employed to identify the ways leading to the occurrence of CDE. Without quantitative analysis, this method fails to show the risk level of the system in intuitive number and provides us with rough understanding of the safety status of the system. Besides, pre-cautionary measures and some important measures cannot be put forward and the employed measures are not aimed at the outstanding problems as well as potential risks. What’s more, the safety checklist analysis fails to aggregate the judgement of experts to reach a consensus, which makes the judgements more subjective. By comparison, the novel method proposed in this paper can overcome the above problems in the safety checklist, which is more scientific, objective and efficient.

## Conclusions

This paper proposed a novel method for a fuzzy fault tree associated with the VB program to identify and assess the risks of CDE. Conclusions can be drawn as follows:

- The CDE fault tree constructed in this paper can reflect the CDE process comprehensively, in which the influence of gas concentration and fugitive coal dust that are overlooked easily is taken into consideration. Additionally, the CDE fault tree can help us identify the potential causes and determine the various paths which easily lead to CDE.
- With the application of the fuzzy set theory and expert elicitation into conventional FTA, the exact value of the IOBE can be obtained, and the quantitative analysis of the CDE fault tree can be reasonably carried out. Synthetically relative deviation distance techniques make this quantitative analysis more effective.
- The VB program technique remarkably simplifies the process of analysis and can work out the calculation results in a few minutes. Due to the complexity of the coal mine system, the fuzzy fault tree analysis of CDE is very difficult and time-consuming. The VB program technique makes it possible to quickly solve the complex fault tree for CDE.
- This novel method was successfully applied to assess the CDE risk in the Zhuxianzhuang Coal Mine and some valuable comments and suggestions are provided to the decision makers. The results have verified the effectiveness of this method. Therefore, it is believed that this method can be used for CED accident prevention and protect the safety of workers.
